# Long term trends at a comprehensive cancer center during the COVID‐19 pandemic

**DOI:** 10.1002/cnr2.1853

**Published:** 2023-06-25

**Authors:** Emily C. Chow, Nicholas D. Sandercott, Olivia E. Yoo, Carolyn Coyle, Jared Johnson, Michael T. Chung, George H. Yoo

**Affiliations:** ^1^ Department of Otolaryngology‐Head and Neck Surgery Karmanos Cancer Institute & Wayne State University Detroit Michigan USA; ^2^ Department of Oncology Karmanos Cancer Institute & Wayne State University Detroit Michigan USA

**Keywords:** COVID‐19, telehealth, telemedicine

## Abstract

**Background:**

As the ongoing public health crisis from Coronavirus Disease 2019 (COVID‐19) pandemic puts strains on current models of cancer care, many health care centers had to adapt to minimize the risk of exposure and infection. The effects of the COVID‐19 pandemic in a comprehensive cancer center were determined.

**Aims:**

To measure the impact of the COVID‐19 pandemic on care delivery at a comprehensive cancer center.

**Methods:**

The number of on‐site and telehealth visits (TH) were obtained from scheduling software. Multiple factors including total visits, telehealth visits, screenings for cancer diagnosis, and cancer treatments were tracked from 2 years before the pandemic onset through 2022. The length of stay (LOS) and Case Mix Index (CMI) were calculated using hospital database.

**Results:**

In the third quarter of FY 2020, telehealth visits (TH) represented a fifth of total patient encounters. Cancer treatments, such as chemotherapy, radiation therapy, and surgery, decreased during the pandemic with number of surgeries being most affected (23% decrease in 2020 compared to the previous fiscal year). The average length of stay (LOS) was also longer with less discharges per given time during the pandemic. The increased LOS was related to increased severity of patient illnesses since CMI was higher. Screening mammograms decreased to a nadir of 58% in 2021 as compared to those screened in pre‐pandemic fiscal years.

**Conclusion:**

The COVID‐19 pandemic impacted many aspects of care, such as treatment and screenings. Many of these factors had to be postponed due to the fear of acquiring COVID‐19 and access to care. The findings presented implicate that the delays and changes in cancer care during the pandemic resulted in less screening and treatment of more advanced disease.

## 1 INTRODUCTION

In late 2019, China had reported rapidly emerging clusters of pneumonia cases caused by severe acute respiratory syndrome coronavirus 2 (SARS‐CoV‐2), prompting the World Health Organization to declare Coronavirus disease (COVID‐19) a global pandemic.^1^ Before the distribution of COVID‐19 vaccinations became commonplace, great effort and focus was placed on preventing the spread of infection, especially in the context of treating cancer patients. The Center of Disease Control issued mandates urging mask wearing and the practice of social distancing to deter the spread of infection. Likewise, the practice of telehealth, defined as the interactions among patients and providers through telephone, e‐mail, video chats, and the Internet, became an increasingly popular route of communication.^2^

Many patients avoided hospitals and healthcare settings where many cases were documented, thus risking delayed detection of cancer diagnoses and treatment. This general avoidance of healthcare ultimately places patients at a greater risk for receiving an overall poorer prognosis due to the likeliness of them presenting with a more advanced cancer stage.^3^ Even when the severity of the pandemic lessened did the utilization of cancer care still lag behind pre‐pandemic states, and the lasting impact of cancer progression, morbidity, and mortality remains unclear.^4^ While the associated risks stemming from a worldwide pandemic are still being evaluated, we sought to further elucidate how the COVID‐19 pandemic affected cancer screening and treatment at a comprehensive cancer center in southeast Michigan.

## 2 MATERIALS AND METHODS

During the COVID‐19 pandemic, the total visits and telehealth visits (TH) in a comprehensive cancer center clinic were measured. A visit was a TH if video conferencing or audio call were used to complete and document a patient interaction. The number of on‐site and telehealth visits were obtained using the scheduling software. The number of radiology visits, discharges, screening mammograms, surgical cases, chemotherapy, and radiation treatments during this timeframe were also measured.

The length of stay (LOS) was calculated by total hospital days divided by the number of discharges for the time period. The Case Mix Index (CMI) is a measure to determine the complexity and severity of patient illnesses that is weighted for each type of discharge. CMI is calculated by summating the relative Medicare Severity Diagnosis Related Group (MS‐DRG) weight for each discharge and dividing this by the total number of Medicare and Medicaid discharges for a period of time.^5^ The average data for FY 2017, 2018 and 2019 were measured and compared as a ratio to FY 2020, 2021 and 2022. These data were organized by fiscal year (FY) which is October through September and further subdivided by quarter (Q1. October–December; Q2. January–March; Q3. April–June; Q4. July–September). The primary outcome measured in this study is the impact of the COVID‐19 pandemic on healthcare volumes with a secondary outcome measured as the effect of the pandemic on the number of breast cancer screenings performed.

## 3 RESULTS

**FIGURE 1:**
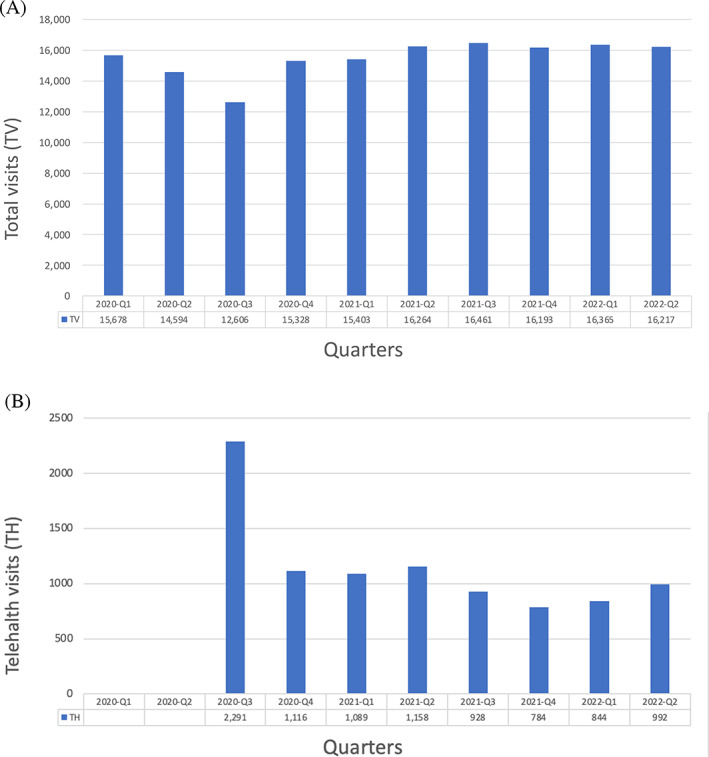
(A) Total clinic visits (TV) and (B) telehealth visits (TH) for fiscal years (FY) 2020, 2021 and 2022 (YTD) by quarters. Fiscal year starts on October and ends in September. TH were first offered in April 2020.

In the beginning months of the pandemic, cases and deaths from COVID‐19 began to skyrocket, prompting the Karmanos Cancer Center to allow only essential visits, such as cancer treatments and diagnosis.^6^ Many other visits, such as those for cancer surveillance, had to be delayed or converted to telehealth to reduce the risk of viral transmission. The largest drop in the number of total clinic visits (TV) at the Karmanos Cancer was observed from the second quarter of FY 2020 to the third quarter of FY 2020, resulting in a loss of 1988 total visits (Figure 1). This drop correlates with the timeline in which the state of Michigan declared a state of emergency at the end of March 2020 and is reflected in the lowest recorded total number of visits at 12606 visits from March to May of 2020. Telehealth visits (TH) were first introduced in April 2020 with a peak of 2291 visits in the third quarter of FY 2020. This represented 18.2% of all total visits, thus telehealth visits composed of nearly a fifth of all total visits at the Karmanos Center. The number of telehealth visits recorded for the subsequent quarters (2020–4, FY 2021, 2022–1, 2022–2) declined dramatically to an average of 987 telehealth visits, which is a decrease of 57% from the third quarter of FY 2020. A rise in total number of visits from the third quarter to the fourth quarter of FY 2020 was witnessed at an increase of 2722 total visits, and the subsequent total visits remained steady at an average of 16 151 total visits (FY 2021, 2022–1, 2022–2). Telehealth visits during this time were still conducted, although they only made up 6.1% of all total visits in the second quarter of 2022. The number of telehealth visits peaked when first introduced in FY 2020 (N = 1704) and declined in subsequent years (Table 1). In FY 2021 and FY 2022, the total number of visits and telehealth visits remained steady (Table 1).

**TABLE 1:** In fiscal years (FY) 2020, 2021 and 2022 (YTD), quarterly average total clinic visits (TV) and telehealth visits (TH). Fiscal year starts on October and ends in September. TH were first offered in April 2020.

	FY19	FY20	FY21	FY22
TH		1704	990	918
TV	15 998	14 552	16 080	16 291
TH/TV		11.7%	6.2%	5.6%

**FIGURE 2:**
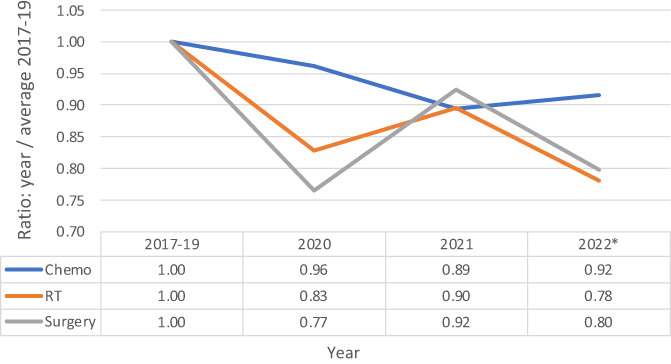
The ratios of chemotherapy (Chemo), radiation treatments (RT) and surgery for fiscal years (FY) 2020, 2021 and 2022 (YTD) as compared to FY 2017–2019 averages. Fiscal year starts on October and ends in September.

The pandemic effects on cancer treatments were compared to pre‐pandemic levels. Compared to the FY 2017–2019 averages (Figure 2), chemotherapy treatments were slightly lower; ratios of 0.96, 0.89, and 0.92 in FY 2020, FY 2021, and FY 2022, respectively, while radiation therapy and surgery had more dramatic initial declines. Radiation therapies saw a decline at ratios of 0.83, 0.90, and 0.78 in FY 2020, FY 2021, and FY 2022, respectively. The ratios of surgery followed a very similar pattern with ratios of 0.77, 0.92, and 0.80 in FY 2020, FY 2021, and FY 2022. All three treatment regimens did not return to the FY 2017–19 averages. Since surgery and radiation are used in early‐stage disease and chemotherapy is added for advanced disease, one assumption is that patients with more advanced disease are being treated in this time frame.

**FIGURE 3:**
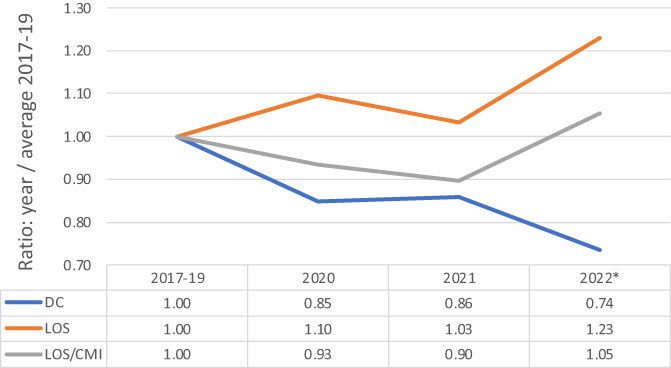
The ratios of number of discharges (DC), length of stay (LOS) and LOS/case mix index (LOS/CMI) for fiscal years (FY) 2020, 2021 and 2022 (YTD) as compared to FY 2017–2019 averages. Fiscal year starts on October and ends in September.

During the pandemic, inpatient length of stay (LOS) increased from FY 2017–19 (Figure 3). The number of discharges from FY 2020, 2021, and 2022 declined from the FY 2017–19 with ratios of 0.85, 0.86, and 0.74. Since the LOS/CMI remained relatively unchanged in FY 2020, 2021, and 2022 from FY 2017–19, the increased LOS is related to higher CMI. In terms of the ratio of LOS/CMI, in FY 2020, the ratio of LOS/CMI decreased by 7% compared to FY 2017–2019. In the background of LOS increasing by 10% during this same time‐period, the increase in ratio can be explained by an even greater increase in CMI, denoting an increase in disease and diagnosis severity. A similar trend is seen in FY 2021, even if the average LOS decreased slightly by 7% and the CMI decreasing by 3% compared to FY 2020. This may be attributed to there being overall sicker patients who required more intense and comprehensive care due to possible COVID‐19 infection.

**FIGURE 4:**
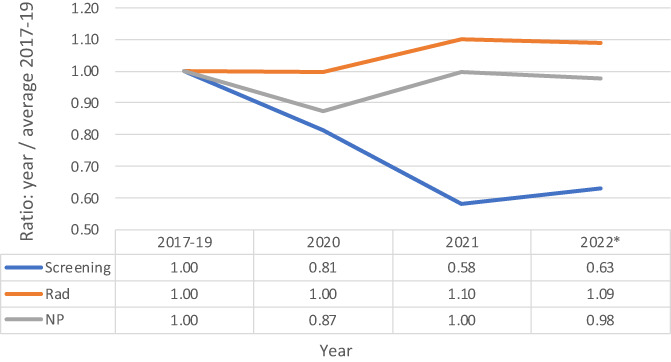
The ratios of number of screening mammograms, radiology visits and new patients (NP) for fiscal years (FY) 2020, 2021 and 2022 (YTD) as compared to FY 2017–2019 averages. Fiscal year starts on October and ends in September.

The number of radiology studies (excluding breast screening) were identical in FY 2020 and increased in FY 2021 to FY 2022 (Figure 4). The number of screening mammograms drastically decreased in FY 2020 and FY 2021 by 9% and 42%, respectively, and remained lower in FY 2022 (Figure 4). The new patients declined 13% in FY 2020 from FY 2017–19; however, new patients rebounded to normal levels in FY 2021 and FY 2022 (Figure 4).

## 4 DISCUSSION

With the COVID‐19 pandemic came many concerns regarding its impact on patients with cancer. This concern stems from the fact that many cancer patients delayed consultations for initial diagnosis and treatment, perhaps due to reluctance from many primary care services to make referrals during the height of the pandemic.^7^ These findings are reflected in the decrease in the number of new patients during FY 2020 compared to FY 2017–19 at this cancer center (Figure 4). The COVID‐19 pandemic also evoked a substantial amount of fear and anxiety in patients that lead them to delay treatment, which jeopardized adherence to therapy.^8^ Based off these findings, it may be inferred that delays in care could lead to worse outcomes in terms of mortality and treatment options, especially in a complex patient population where timing of treatment is of utmost importance.

To balance on‐time care with the risk of exposure to COVID‐19, many institutions established the use of telehealth medicine, which consisted of nearly one fifth of total clinic visits at the Karmanos Cancer Center at the pandemic onset (Figure 1). Its main purpose was to provide a feasible alternative to reduce chances for contact and spread of the virus, and its use in the cancer care setting allowed for surveillance and follow‐up in this population.^9^ At the height of the pandemic in FY 2020‐Q3, the surge in telehealth visits as compared to the lowest‐recorded total number of visits should be attributed to the fact that many healthcare encounters were not conducted in person due to the risk of infection. The decline in telehealth usage, however, in subsequent years compared to when it was first introduced may be attributed to it not having yet entered a mature phase of integration into cancer care.^10^ Even so, the use of telehealth medicine was adopted into an even more integral role in the cancer care journey in many institutions given its many advantages, such as cost savings, decreased travel time, and easy access.^11^ This is perhaps why, even if there was an appreciable decline in the percentage of telehealth encounters that made up total clinic visits at the Karmanos Center, many institutions still offered this alternative platform to continue giving much needed healthcare to their patients.

Given the significance of increased mortality associated with cancer and COVID‐19 infection and the consequence of delayed cancer diagnosis, screenings for breast cancer malignancies should have theoretically increased, but its exact opposite was observed (Figure 4). Specifically, the numbers of screening mammograms for detecting breast cancer have dropped nearly 40% from FY 2017–19 averages to FY 2021, reflecting a risk for missing many early‐stage breast cancers that are more easily treatable than later‐stage, potentially disseminated, cancers. This begs an even larger question of what could have been done differently from a public health standpoint in balancing continued screening services amidst a dangerous, high‐risk environment that the pandemic posed. This persistent decline in mammograms raises many concerns because late detection and treatment of breast cancer leads to more advanced stages that are more difficult to treat and are associated with higher mortality.^12^

In the context of this study, overall declining trends in all cancer therapies were noted, although these treatment modalities were not all affected equally. Compared to radiation therapy and chemotherapy, the pandemic onset impacted surgery the most, with a decline of 23% in FY 2020 compared to FY 2017–19. One possible explanation is that some surgeons delayed excisional surgery in less aggressive cancers compared to those with highly aggressive malignancies.^13^ Another, perhaps more likely, explanation is that the benefit of postponing surgical therapy to reduce likely infection outweighed the benefit of excising the cancer itself. Regardless of the reasons, surgeries continued to decline and never made it back to pre‐pandemic numbers. More advanced cancers tend to utilize more inpatient hospital stays because of complications of cancer treatment and tumor progression. These data show that LOS increased during the pandemic, which was correlated to sicker patients with a higher CMI. Although these trends were observed, it is important to note a limitation in study sample size as well as a lack of comparative information on different types of cancer from the pre‐ and post‐pandemic timeline. Further investigation should seek to elucidate the impact of these observed trends in cancer care for this patient population.

## 5 CONCLUSION

The COVID‐19 pandemic impacted many aspects of care, such as treatment and screenings. Many of these factors had to be postponed due to the fear of acquiring COVID‐19 and access to care. The findings presented implicate that the delays and changes in cancer care during the pandemic resulted in less screening and treatment of more advanced disease.

## AUTHOR CONTRIBUTIONS

All authors had full access to the data in the study and take responsibility for the integrity of the data and the accuracy of the data analysis. Conceptualization, George H. Yoo, Michael T. Chung, and Jared Johnson; Methodology, George H. Yoo, Validation, Emily C. Chow and George H. Yoo, Resources, Emily C. Chow, Nicholas D. Sandercott, Olivia E. Yoo, and Carolyn Coyle, Data Curation, Emily C. Chow, Nicholas D. Sandercott, George H. Yoo, Writing – Original Draft, Emily C. Chow and Nicholas D. Sandercott, Writing – Review & Editing, Emily C. Chow, Nicholas D. Sandercott, Olivia E. Yoo, Carolyn Coyle, Michael T. Chung, Jared Johnson, and George H. Yoo, Visualization, Emily C. Chow and George H. Yoo.

## CONFLICT OF INTEREST STATEMENT

The authors have stated explicitly that there are no conflicts of interest in connection with this article.

## ETHICS STATEMENT

This study is exempted from IRB approval through Paragraph 4 Part (ii) regarding secondary research involving non‐identifiable human subjects.

## Data Availability

The data that support the findings of this study are available on request from the corresponding author. The data are not publicly available due to privacy or ethical restrictions.
